# FMRI investigation of cross-modal interactions in beat perception: Audition primes vision, but not vice versa

**DOI:** 10.1016/j.neuroimage.2010.09.033

**Published:** 2011-01-15

**Authors:** Jessica A. Grahn, Molly J. Henry, J. Devin McAuley

**Affiliations:** a MRC Cognition and Brain Sciences Unit, Cambridge, UK; b Department of Psychology, Bowling Green State University, Bowling Green, OH, USA; c Department of Psychology, Michigan State University, East Lansing, MI, USA

**Keywords:** Timing, Temporal processing, Rhythm perception, Cross-modal processing, fMRI, Auditory perception, Visual perception

## Abstract

How we measure time and integrate temporal cues from different sensory modalities are fundamental questions in neuroscience. Sensitivity to a “beat” (such as that routinely perceived in music) differs substantially between auditory and visual modalities. Here we examined beat sensitivity in each modality, and examined cross-modal influences, using functional magnetic resonance imaging (fMRI) to characterize brain activity during perception of auditory and visual rhythms. In separate fMRI sessions, participants listened to auditory sequences or watched visual sequences. The order of auditory and visual sequence presentation was counterbalanced so that cross-modal order effects could be investigated. Participants judged whether sequences were *speeding up* or *slowing down*, and the pattern of tempo judgments was used to derive a measure of sensitivity to an implied beat. As expected, participants were less sensitive to an implied beat in visual sequences than in auditory sequences. However, visual sequences produced a stronger sense of beat when preceded by auditory sequences with identical temporal structure. Moreover, increases in brain activity were observed in the bilateral putamen for visual sequences preceded by auditory sequences when compared to visual sequences without prior auditory exposure. No such order-dependent differences (behavioral or neural) were found for the auditory sequences. The results provide further evidence for the role of the basal ganglia in internal generation of the beat and suggest that an internal auditory rhythm representation may be activated during visual rhythm perception.

## Research Highlights

►Perception of a beat occurs more easily with auditory than visual temporal sequences. ►Pre-exposure to auditory sequences enhances visual beat perception. ►Pre-exposure to visual sequences does not alter auditory beat perception. ►Putamen activity predicts strength of visual beat perception.

## Introduction

Understanding of how brains keep time is central to understanding many aspects of perceptual, cognitive and motor function. In this regard, it is notable that although much perceptual information gleaned from the environment is *modality-specific* (i.e., exclusively conveyed through a single sense), temporal information, such as the duration of a stimulus or the temporal pattern of a series of stimuli, is *amodal* in nature (i.e., conveyed by more than one sense). For example, consider the periodic “beat” produced by the repetitive striking of a bass drum; this beat can be heard, seen, or even felt depending on how close one is to the drummer. Thus, an important component of how brains keep time is the issue of how brains manage to successfully combine temporal cues from different sensory modalities to effectively guide behavior. One consideration that has influenced much research at the intersection of time perception and cross-modal processing is whether, despite the amodal nature of time, the auditory system may demonstrate a specialization (and priority) for processing temporal information over other sensory systems (e.g., Fendrich and Corballis, 2001; Guttman et al., 2005; Repp and Penel, 2002). The focus of this article is the neural basis of auditory specialization for one aspect of temporal processing–the perception of a periodic “beat”–with special emphasis on possible cross-modal effects on visual temporal patterns following exposure to auditory patterns with identical temporal structure.

Broad behavioral support for auditory temporal processing dominance comes from a number of sources (Goodfellow, 1934; Kolers and Brewster, 1985; Grondin and Rousseau, 1991; Grondin, 2001; Rencanzone, 2002; Repp and Penel, 2002; Grondin and McAuley, 2009). Of note, in the temporal ventriloquism effect, there is a tendency to perceive a single visual “flash” and a single auditory “click” that are in close temporal proximity as simultaneous, with misperception of the visual stimulus in the direction of the auditory stimulus accounting for most of the shift (Fendrich and Corballis, 2001). Related research has observed an auditory driving effect whereby a repetitive sound (auditory flutter) presented simultaneously with a repetitive visual stimulus (visual flicker) causes the perceived visual flicker rate to shift toward the auditory flutter rate, even though the auditory flutter and visual flicker rates are easily distinguishable (Rencanzone, 2002). Most directly relevant for the present investigation are findings that auditory rhythms, such as the sound of a beating drum, are much more likely to lead to the perception of a periodic beat or pulse than visual rhythms—even when the same temporal information is present (Patel et al., 2005). Auditory rhythms have additionally been found to be better discriminated and remembered than visual rhythms (Glenberg et al., 1989; Glenberg and Jona, 1991; Grondin and McAuley, 2009), and show asymmetric interference effects whereby participants have much more difficulty making judgments about visual rhythms in the presence of to-be-ignored auditory rhythms than they do making the same judgments about auditory rhythms in the presence of to-be-ignored visual rhythms (Repp and Penel, 2002; Guttman et al., 2005).

Despite well-documented auditory–visual behavioral differences in a range of different timing tasks, the neural systems underpinning auditory and visual timing tend to show remarkable overlap (Schubotz et al., 2000; Shih et al., 2009). Commonly activated brain areas for visual and auditory timing tasks include the premotor and supplementary motor cortices, basal ganglia, and cerebellum (Ivry and Keele, 1989; Harrington et al., 1998; Harrington and Haaland, 1999; Schubotz et al., 2000; Schubotz and von Cramon, 2001; Macar et al., 2002; Ferrandez et al., 2003; Nenadic et al., 2003; Coull, 2004) with much of the debate focused on the locus of a central “timekeeper” or clock. A few studies have specifically examined the neural bases of beat perception, but relatively little is known about the specific neural mechanisms mediating modality differences, as most investigations of beat perception have tested only the auditory modality (Jongsma et al., 2004; Grahn and Brett, 2007; Grahn and McAuley, 2009). Moreover, those studies that have compared across modalities have focused on production, rather than perception (Penhune et al., 1998; Jantzen et al., 2005; Karabanov et al., 2009). In the perceptual domain, cerebellar and premotor structures respond strongly to auditory rhythms, regardless of whether beat perception is occurring (Grahn and Brett, 2007; Chen et al., 2008; Bengtsson et al., 2009). The basal ganglia and supplementary motor area (SMA), however, respond more strongly during beat perception than during listening to irregular auditory sequences that lack a steady beat (Grahn and Brett, 2007). SMA activity is also higher in those individuals who behaviorally show greater levels of beat perception in ambiguous sequences (Grahn and McAuley, 2009). Finally, the basal ganglia response is greatest when beat perception is internally generated (for example, when the beat is not heavily emphasized by external features of the auditory stimuli, but rather imposed onto the stimuli by the listener) (Grahn and Rowe, 2009). Thus, the basal ganglia and SMA are important structures for beat perception, with the former playing a greater role in internal generation, although this is only known for the auditory modality.

**Fig. 1:**
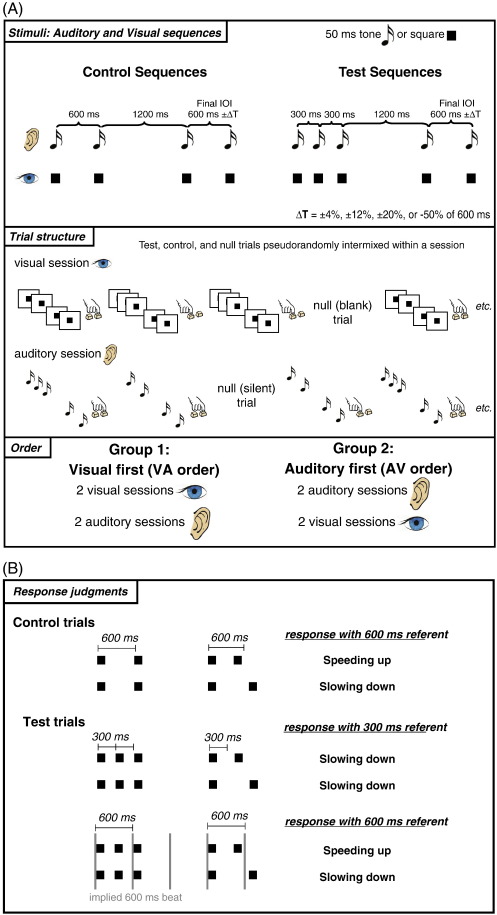
Tempo judgment paradigm: (A) Illustration of control and test sequences and design summary. Control sequences comprised four elements and test sequences comprised five elements. Sequence elements were marked by tones in the auditory condition and squares in the visual condition. Participants were divided into two groups: half received two sessions in the auditory modality followed by two sessions in the visual modality (AV order), the other half received two sessions of visual followed by two sessions of auditory (VA order). Control and test sequences were randomly intermixed within each session. (B) Task and typical pattern of responses to control sequences and pattern of responses to test sequences when the implied beat is perceived and when the implied beat is not perceived. For control sequences, speeding up or slowing down judgments are made relative to the explicitly heard or seen initial 600-ms interval. For test sequences, judgments can be made relative to the explicitly heard or seen 300-ms initial intervals, or to an implied 600-ms interval. The 600-ms referent is only used when individuals perceive a steady “beat” that is implied by the temporal structure of the test sequences (Grahn and McAuley, 2009).

In order to address this gap in knowledge, the present study compared beat perception in the auditory and visual modalities, combining fMRI with a recently developed behavioral paradigm that uses tempo (rate) judgments to assess beat sensitivity (McAuley et al., 2006a; Grahn and McAuley, 2009; McAuley and Henry, 2010; Snyder et al., in press). Stimuli were sequences of discrete elements (tones or flashes), with the timing of the sequence elements chosen to be within a range of rates (~ 600 ms) known to yield reliable beat perception (Parncutt, 1994; Drake et al., 2000; McAuley et al., 2006b).Of central interest was the pattern of *speeding up* versus *slowing down* judgments for ambiguous “test” sequences where the perception of a beat was possible, but for which the beat was not explicitly marked and its perception was not required to perform the task (see Fig. 1). Based on previous research, we expected that observers would be less sensitive to an implied beat in visual sequences than in auditory sequences. Of key interest here, however, was how the order in which participants experienced each modality affected their beat perception. By counterbalancing modality order (either auditory first or visual first), we could examine cross-modal influences. We hypothesized that during the auditory-first condition, participants would create an internal representation that would increase beat perception during the subsequent visual condition. No similar internal representation would be available for those in the visual-first condition, as they would not yet have had exposure to the auditory sequences. Given the previous findings connecting the basal ganglia to beat perception, particularly during internal generation, we predicted greater basal ganglia activity for auditory sequences than for visual sequences, and, moreover, that the basal ganglia response would be greater for visual sequences experienced after the auditory condition than before. Conversely, no modality-dependent order effects on behavior or brain activity were expected for the auditory sequences.

## Materials and methods

### Design

In our design, listeners judged two types of auditory and visual sequences, termed “control” and “test” (shown in Fig. 1). Each of these sequences contained one of seven variable-length final intervals. This resulted in a 2 (modality: auditory, visual) × 2 (sequence type: control, test) × 2 (order: auditory first, visual first) × 7 (final interval: 600 ms ± 4%, ± 12%, ± 20%, −50%) mixed-factorial design. Modality, sequence type, and final interval were manipulated within subjects, while order was a between-subjects factor. A range of final intervals was used in order to construct psychometric curves and measure discrimination thresholds for each subject in each condition.

### Participants

Twenty-seven right-handed neurologically normal volunteers (*n* = 11, male) between the ages of 18 and 48 years (*M* = 28 years, *SD* = 7) participated in exchange for a cash payment. Participants all self reported normal hearing and had a range of formal musical training (*M* = 4.3 years, *SD =* 4.2). Informed consent was obtained from all participants.

### Stimuli

Auditory stimulus sequences were composed of 50-ms sine tones with a frequency of 440 Hz, and were generated by Audacity 1.2.6 for Windows (http://audacity.sourceforge.net/). Visual sequences were composed of black squares appearing for 50 ms on a white background. There were two types of sequences: control and test. For the control (4-element) sequences, the onsets of the elements marked out three time intervals with the following durations: 600 ms, 1200 ms, and a variable final duration of 600 ms ± 4%, ± 12%, ± 20%, or −50%. The test (5-element) sequences were identical except that an additional element was inserted in the middle of the initial 600-ms interval to create two 300-ms intervals; see Fig. 1. The key aspect of the test sequences is that in addition to the pair of 300-ms intervals marked by the first three elements of the sequence, the temporal structure of the sequence can convey to individuals the sense of a periodic beat at regular 600-ms interval intervals (Povel & Essens, 1985). In the test sequences, the initial 600-ms inter-beat-interval is marked by the onsets of the first and third elements, which introduces a missing beat halfway through the 1200-ms gap so that the first onset of the final interval is ‘on the beat’. From this perspective, the final element of the test sequences is always ‘early’ or ‘late’ with respect to the expected beat. Test sequences could also be judged on the basis of the initial 300-ms intervals, in which case the final interval will generally be heard as slowing down (as most of the variable final intervals were longer than 300 ms). Therefore, trials where the final interval was 300 ms (600 minus 50%) were included so that individuals who generally heard or saw test trials with the other final intervals as *slowing down* could not prepare their response earlier than individuals who judged test trials as either *slowing down* or *speeding up*. Test and control sequences did not differ in total duration.

### Procedure

In separate sessions, participants were presented with either auditory or visual sequences and judged whether at the end of the sequence, they felt the sequence was *speeding up* or *slowing down*. Responses were made using a button box positioned under the right hand; the middle finger was used to indicate the sequence was speeding up and the index finger was used to indicate the sequence was slowing down. We emphasized to participants that we were simply interested in their impressions of the sequences and that it was acceptable for them to respond that all of the sequences were *speeding up*, that all of the sequences were *slowing down* or that they were sometimes *speeding up* and other times *slowing down*. Participants were not shown a diagram of the task or told anything about the sequences other than the number of elements.

Prior to scanning, participants completed eight familiarization trials outside of the scanner. Each participant completed four scanning sessions (two auditory and two visual). Participants were randomly assigned to receive either the two visual sessions followed by the two auditory sessions (VA order), or vice versa (AV order). Within a session, participants completed 80 trials in total, and the sequence types were presented in random order. For each sequence type, there were 5 trials of each final interval except for the −50% interval, for which there were 10 trials. Participants were given 2.5 s to respond, which was followed by an inter-trial interval of 1 s. Nine “null” events, 4.5 s long, were randomly interspersed in each session in order to resolve the hemodynamic response in analysis.

### Image acquisition and preprocessing

Participants were scanned in a 3T Siemens Tim Trio using a head coil gradient set. To ensure participants were comfortable, foam pads were placed around the head and supported the legs. Auditory stimuli were presented over headphones; attenuation of scanner noise was achieved with insert earplugs rated to attenuate by ~ 30 dB (3 M 1100 earplugs, 3 M United Kingdom PLC, Bracknell, UK). The participants also wore ear defenders. When wearing earplugs and ear defenders, none of the participants reported difficulty in hearing the stimuli or focusing on the task. Visual stimuli were projected to a mirror positioned over the participant's head. Participants were instructed not to move any part of their body during the scan other than to respond. Button press responses were recorded with millisecond accuracy.

Functional data were collected using a gradient echo echo-planar imaging (EPI) sequence (36 slices, slice thickness 3 mm + 0.75 mm inter-slice gap, matrix size of 64 × 64, in plane resolution 3 × 3 mm, total FOV = 19.2 × 19.2 cm, TE = 30 ms, TR = 2.19 s, flip angle = 78°). 260 volumes were collected in each session (total duration ~ 9.5 min). These EPI parameters enabled whole-brain coverage, including the cerebellum, for all participants. High-resolution 1 × 1 × 1 mm MPRAGE anatomical images were collected for anatomic localization and coregistration. SPM5 was used for preprocessing and analysis (SPM5; Wellcome Department of Cognitive Neurology, London, UK). Images were slice-timing corrected, then realigned spatially (to correct for motion) to the first image in the series, using a least squares approach with 6 rigid-body parameters, and trilinear interpolation. The MPRAGE image was segmented and normalized (using affine and smoothly nonlinear transformations) to a brain template in Montreal Neurological Institute (MNI) space. The resulting normalization parameters were applied to the coregistered EPIs and EPI images were smoothed with an 8 mm full-width half-maximum Gaussian kernel.

### Data analyses

#### Behavioral data

For each participant, proportions of *speeding up* responses were calculated for each of the 12 trial types (2 sequence types X 6 final intervals) in the auditory and visual modalities. A signal detection model was then fit to these values to obtain two measures for each participant in each modality: a threshold estimate that assessed temporal discrimination ability and an index of beat sensitivity, *w*: see supplementary material for full model details. In the model, it is assumed that participants compare the final interval to one of two temporal referents: a 300-ms referent corresponding to the explicit time interval marked by the initial three elements of the test sequences and a 600-ms referent corresponding to the implied beat. Previously, Grahn and McAuley (2009) showed that individuals vary in the extent to which they rely on the 300-ms and 600-ms temporal referents when making *speeding up* / *slowing down* judgments about the test sequences in the auditory modality. The value of the beat sensitivity index *w* ∈ [0, 1] indicates the relative weighting of the two temporal referents. Larger values of *w* indicate greater sensitivity to the implied 600-ms referent (beat) than smaller values of *w,* with a *w* of zero corresponding to complete reliance of tempo judgments on the explicit 300-ms referent. For the control sequences, values of *w* are expected to be close to 1.0 for most participants as the 600-ms temporal referent is explicitly marked by the initial two sequence elements (and the 300-ms referent is not present). For test sequences, however, both a 300-ms referent (explicit) and a 600-ms referent (implicit) could be used. Judgments made on the basis of the 600-ms referent with the test sequences demonstrate sensitivity to the implied beat. Here, our primary interest in this index is not in using it to assess individual differences, but rather to use it to examine potential modality differences in beat sensitivity. That is, do *w*-values differ between auditory and visual modalities?

#### FMRI data

Stimuli were modeled using a regressor made from an on-off boxcar convolved with a canonical hemodynamic response function. The length of the boxcar function corresponded to the time between the onset of the first stimulus in a trial and the offset of the last stimulus. Button presses were modeled with a delta function convolved with a canonical hemodynamic response function. EPI volumes with more than 4 mm movement in any plane were included as covariates of no interest to minimize movement artifacts. Low-frequency noise was removed with a 128 s high-pass filter. Results estimated from single-subject models were entered into second-level random effects analyses for standard SPM group inference (Penny and Holmes, 2003).

For each participant, contrast images were generated comparing test and control sequences in both modalities to the implicit baseline. These were then entered into a second-level random effects analysis using a mixed-measures analysis of variance with three factors: modality (auditory, visual), order of presentation (AV order, VA order), and sequence type (test, control). Specific contrasts of interest included: auditory condition versus visual condition, a conjunction of the auditory and visual conditions, test sequences versus control sequences, and interactions between order and modality (differences in the visual condition between the AV and VA orders, and differences in the auditory condition between the AV and VA orders).

## Results

### Behavioral results

#### Discrimination thresholds

Initial inspection of the behavioral data revealed four participants who showed atypical performance on control sequences with very poor tempo discrimination of visual control sequences only. The average relative just-noticeable difference in tempo (JNDs) for visual control sequences for these participants was 45.0% ± 22.2%. Analyses of the behavioral data were run both with and without these participants in order to assess their potential contribution to the pattern of results. No substantive differences were found in the two sets of analyses. Nonetheless, to be conservative, the four participants with very poor visual tempo sensitivity were excluded from the analyses reported below to avoid exaggerating any modality differences in beat perception that would be due simply to some participants not being able to reliably detect changes in the visual tempo. Without these participants, relative JNDs in tempo were still modestly higher for visual sequences (*M =* 19.3% ± 2.5%) than for auditory sequences (*M* = 10.1% ± 0.7%), *t*(22) = 3.87, *p* < 0.01), but critically the remaining participants were clearly able to detect tempo changes in both modalities. Relative JNDs for auditory sequences were somewhat lower for the VA order (*M =* 9.15% ± 0.41%) than for the AV order (*M =* 12.23% ± 2.07%), *t*(21) = 2.10, *p* < 0.05; however, relative JNDs for visual control sequences were not different between orders (*M*_*VA*_ *=* 18.48% ± 2.32%; *M*_*AV*_ = 21.16% ± 6.37%), *t*(21) = 0.50, *p* = 0.63.

#### Beat sensitivity index, w

**Fig. 2:**
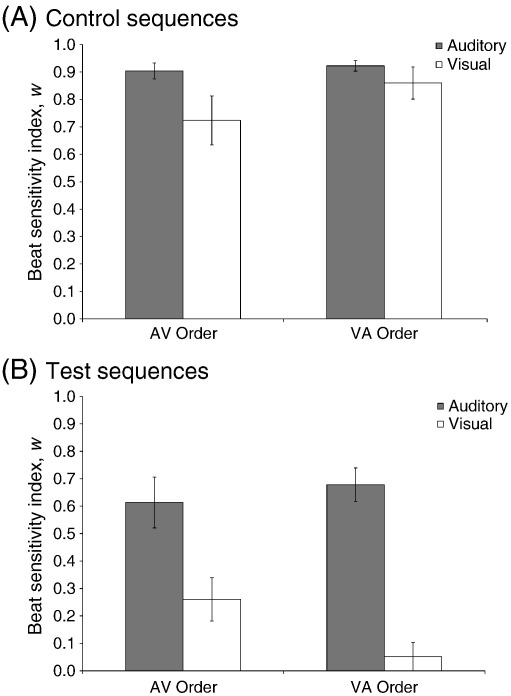
Average values of the beat sensitivity index, *w,* for AV and VA orders for auditory and visual modalities; *w* values are shown separately for control (Panel A) and test (Panel B) sequences. For control sequences, *w* values are not affected by order. For test sequences, there is a significant interaction between modality and order, caused by an increase in *w* for the visual condition in the AV order relative to the VA order. Error bars represent standard error.

Fig. 2 shows average values of *w* (beat sensitivity) for auditory and visual sequences (control and test) for the VA order (Panel A) and the AV order (Panel B). A 2 (modality) × 2 (sequence type) × 2 (order) mixed-measures ANOVA on *w* revealed significant main effects of sequence type, *F*(1, 21) = 80.1, *MSE =* 0.04, *p* < 0.001, η^2^_p_ = 0.79, and modality, *F*(1, 21) = 45.1, *MSE =* 0.04, *p* < 0.001, *η*^2^_p_ = 0.68, a significant modality × sequence type interaction, *F*(1, 21) = 37.3, *MSE =* 0.02, *p* < 0.001, η^2^_p_ = 0.64, and a significant three-way modality × sequence type × order interaction, *F*(1, 21) = 10.5, *MSE =* 0.03, *p* < 0.01, η^2^_p_ = 0.33. Values of *w* for control sequences were predicted to be close to 1 since a 600-ms temporal referent is explicitly marked. As expected, values of *w* were closer to 1 for control sequences (*M* = 0.85 ± 0.03) than for test sequences (*M* = 0.40 ± 0.04), indicating that tempo judgments were more likely to be based on the 600-ms referent. With respect to modality, beat sensitivity was much lower for visual sequences (*M* = 0.47 ± 0.04) than for auditory sequences (*M* = 0.78 ± 0.03), especially for the test sequences where the 600-ms referent was only implied by the temporal structure of the sequence. However, effects of modality were also influenced by the presentation order of the auditory and visual sequences. In general, participants picked up on the implied beat of the visual test sequences more when they had prior auditory exposure (AV order, *M* = 0.26 ± 0.07) compared to no prior auditory exposure (VA order, *M* = 0.05 ± 0.05), *t*(21) = 2.2, *p* < 0.05. However, prior visual exposure did not affect sensitivity to the implied beat of the auditory test sequences (AV order, *M* = 0.61 ± 0.09; VA order, *M* = 0.68 ± 0.06), *t*(21) = −0.6, *p* = 0.57. For the control sequences, in which the beat is explicitly marked, beat sensitivity was high across modalities in both groups of participants (see Fig. 2).

Finally, we considered potential correlations between discrimination thresholds, years of musical training, and the beat sensitivity index, *w*. Correlational analyses revealed that there was no relationship between JNDs and sensitivity to the implied beat of the test sequences for either the auditory sequences, *r*(21) = −0.17, *p* = 0.45 or the visual sequences, *r*(21) = −0.26, *p* = 0.26. Moreover, years of musical training was not correlated with auditory, *r*(21) = − 0.03, *p* = 0.91, or visual JNDs, *r*(21) = 0.02, *p* = 0.95. Musical training was also not correlated with beat sensitivity for either auditory, *r*(21) = 0.12, *p* = 0.60, or visual test sequences, *r*(21) = −0.34, *p* = 0.11.

### fMRI results

#### Effects of modality

The contrast of auditory stimuli (test and control sequences) minus visual stimuli activated several areas, including bilateral superior temporal gyri, basal ganglia, hippocampi, cuneus, right inferior frontal cortex, and insula at a whole-brain corrected (pFDR < 0.05) level of significance. Visual stimuli (test and control sequences) minus auditory stimuli activated bilateral occipital cortex, fusiform gyri, cerebellum, superior parietal cortex, and right inferior frontal operculum at a whole-brain corrected (pFDR < 0.05) level of significance (maxima are reported in Table 1).

#### Effects of task

**Fig. 3:**
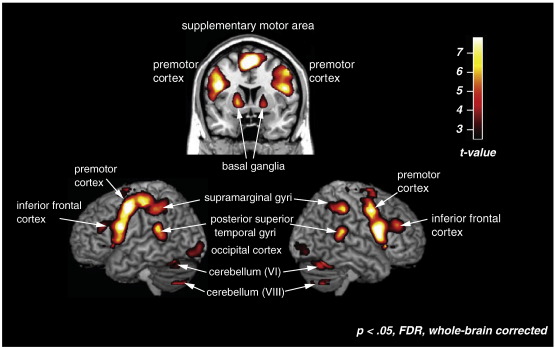
Areas that were significantly active in the conjunction of auditory-rest and visual-rest, pFDR < 0.05, whole-brain corrected.

The auditory–rest and visual–rest contrasts were subjected to a conjunction analysis to find areas significantly activated in both modalities (pFDR < 0.05, conjunction null hypothesis). This analysis showed significant activity in the bilateral inferior frontal cortex (BA 44), SMA, premotor cortex, superior temporal gyri, supramarginal gyri (BA 40), putamen, cerebellum, and thalamus (Table 2 and Fig. 3).

#### Effects of sequence type

Overall, an F contrast of control versus test sequences showed no areas with significant activity differences. Conducting separate F contrasts for each modality still showed no areas with significant activity differences between test and control sequences.

#### Effects of order

The AV order group showed significantly greater activity in the inferior frontal cortex (*t* = 5.85, pFDR < 0.01; *x* = 60, *y* = 15, *z* = 9, BA 44/45) than the VA order group, across all conditions. No areas were significantly more activated in the VA order group compared to the AV order.

#### Interactions

**Fig. 4:**
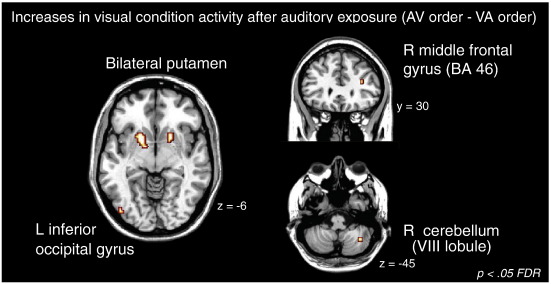
Brain areas with significantly greater activity during the visual condition in the auditory-first order compared to the visual-first order (auditory-first visual condition–visual-first visual condition) at pFDR < 0.05, whole-brain corrected. The image is masked by the F-contrast of the interaction between modality and order at *p* < 0.001 uncorrected to exclude any non-specific order-related differences that were present in both modalities; *y* and *z* indicate position in stereotaxic coordinates.

**Fig. 5:**
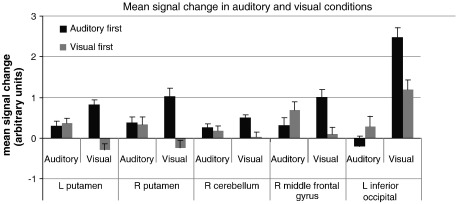
Mean signal intensities extracted for the brain areas showing significantly greater activity during the visual condition in the auditory-first order compared to the visual-first order.

There were no areas exhibiting significant interactions between sequence type (test, control) and order (AV, VA), or between sequence type and modality (auditory, visual). However, several areas did show a significant interaction between modality and order, with larger clusters in the bilateral basal ganglia, and smaller areas of activity in the right anterior insula, left SMA, right inferior temporal gyrus, and left inferior occipital gyrus (see Table 3 for maxima). To determine the nature of the interaction, *t*-tests were performed. For each *t*-test, whole-brain corrected results were inclusively masked by areas significantly activated in the interaction contrast at an uncorrected *p* value of *p* < 0.001. The masking conservatively restricts the *t*-test analysis to brain areas in which an interaction also exists. The *t*-test results showed that activation in the visual condition was influenced by whether it preceded or followed the auditory condition. Specifically, in the visual condition, significantly greater activity was observed for the AV order relative to the VA order (AV visual condition–VA visual condition) in the bilateral putamen, right middle frontal gyrus (BA 46), right inferior temporal gyrus (BA 37), left inferior occipital gyrus (BA 19), and right cerebellum (lobule VIII) (see Table 4 and Fig. 4). The bilateral putamen clusters were of sufficient size to confidently rule out the possibility of false positives, however, the other activation clusters were small (1–6 voxels), and therefore could not be confidently ruled out as false positives (as each cluster was less than 5% of the total number of activated voxels in the contrast, and a pFDR < 0.05 threshold was used). Mean signal intensity was extracted from each activated region to further illustrate the nature of the significant activity differences between modality and order (Fig. 5). Again, no significant visual condition activity increases were observed for the VA order–the AV order (VA visual condition–AV visual condition). Moreover, when activity in the auditory condition for the AV order was compared to the VA order, no significant differences were found (i.e., the neural response was not significantly different in the auditory condition when it was completed before the visual condition versus after the visual condition).

**Fig. 6:**
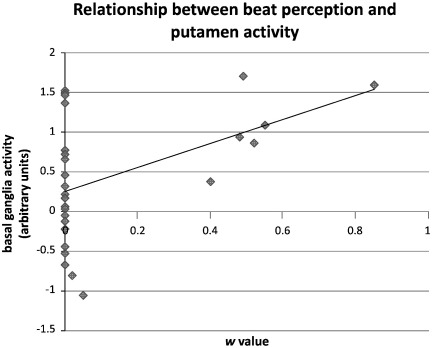
A scatterplot showing average bilateral putamen activity and *w* values from the visual session during fMRI.

To determine whether visual beat sensitivity was directly related to activity increases in the putamen and the right cerebellum during the visual sessions, regression analyses were performed using putamen and cerebellar activity to predict *w* values for visual test sequences. Activity in the left and right putamen was highly correlated (*r*(27) = 0.86, *p* < 0.001) so activity across both putamen regions was averaged together to create a single regressor. This, and average activity in the right cerebellar region, was entered into a multiple regression analysis predicting visual test *w*. Putamen activity was found to significantly predict *w* values, *β =* 0.46, *t(27) = 2.59, p <* 0.016, but right cerebellar activity did not *β* = 0.17, *t(27)* = 0.91*, p =* 0.37*.* A scatterplot showing the relationship between putamen activity and *w* value is shown in Fig. 6. One caveat is that the activated right cerebellar region was only composed of 4 voxels, so the estimate of average activity was likely noisier than the average across the larger putamen clusters.

#### Effects of time in scanner

Because the visual condition was experienced earlier in the scanning experiment by the VA order group, and later in the experiment for the AV order group, the potential for a confound with time in the scanner exists. This possibility was explored with additional analyses to determine if the visual condition differences between the groups were attributable to the effects of time rather than order. A time confound is unlikely to be the explanation, as the two groups *also* experienced the auditory condition at different times, and therefore differences arising solely from time in the scanner would be expected between groups in the auditory condition. Our previous analyses of the auditory VA order–auditory AV order contrast did not find any significant differences between orders in the auditory condition. However, subthreshold differences could exist, so mean signal intensity was examined in each activated region for the two groups. If the simple passage of time accounts for the observed activity increases in these areas, then the VA order group should show greater activity during the auditory condition (which occurred second) than the AV order group in these areas. This was not the case (auditory activity in each area for each group is shown in Fig. 5). A final analysis to rule out time confounds exploited the fact that there were two sessions in each modality, which enabled the effects of time to be broken down within each modality. If any observed differences in visual condition activity are due to time in the scanner rather than order, they should be stronger for the second visual session (which occurs later) compared to the first visual session. As shown in Supplementary Figure 1, the activity shows the opposite pattern: the VA order visual condition activity differences are larger in the first visual session than the second visual session, indicating the effects are not the result of increased time in the scanner, and are most parsimoniously explained by the recent exposure to the auditory condition.

#### Correlations with JND

To determine how brain areas that were sensitive to tempo compared to brain areas that were sensitive to the beat, we also conducted whole-brain analyses using JND scores as regressors. Visual JND scores were used as a regressor for the visual 4- and 5-element fMRI contrast images, and auditory JND scores were used as a regressor for the auditory 4- and 5-element fMRI contrast images. No areas that significantly correlated with JND score were found in either modality, even at a very liberal statistical threshold (pFDR < 0.40).

## Discussion

The current study investigated modality differences in beat perception by combining fMRI with a tempo (rate) judgment paradigm that permitted assessment of both temporal discrimination thresholds and beat sensitivity. We consider the behavioral findings first.

### Behavioral results

Tempo discrimination thresholds were slightly lower in the auditory than the visual modality, indicating greater sensitivity to tempo changes in auditory rhythms. This is consistent with previous behavioral studies that have also found superior auditory (versus visual) performance in temporal interval discrimination (Goodfellow, 1934; Llamon and Goldstone, 1974; Grondin and Rousseau, 1991; Grondin, 2001; Grondin and McAuley, 2009), temporal sequence reproduction and discrimination (Gault and Goodfellow, 1938), and synchronization tasks (Kolers and Brewster, 1985; Repp and Penel, 2002).

Tempo judgments in the current study also revealed substantially greater beat sensitivity for auditory than for visual sequences, despite the identical temporal structure of stimulus sequences in both modalities. This finding fits both with intuitive notions that beat perception occurs more readily for listening than for watching, and empirical work indicating the same (Glenberg and Jona, 1991; Patel et al., 2005). A key behavioral finding was that auditory beat sensitivity was unchanged by watching visual sequences, but visual beat sensitivity was significantly higher after listening to auditory sequences. One possible explanation for this is that listening to the auditory sequences made the subsequent visual tempo discrimination easier, which somehow enabled greater beat sensitivity for visual rhythms. However, we found no evidence for this: the tempo discrimination thresholds in the visual modality did not differ between the VA and AV groups, indicating that overall sensitivity to tempo changes in the visual modality was unchanged by prior auditory exposure. In addition, temporal discrimination thresholds did not correlate with beat sensitivity, suggesting that general timing abilities are independent from sensitivity to an implied beat. Instead, we suggest that exposure to auditory rhythms primes an internal representation of a beat, which can then be exploited during visual performance to promote visual beat perception. Our fMRI results, discussed below, support this account.

Although other studies have shown that audition exerts greater influence over vision during temporal processing than vice versa, these studies have mainly considered concurrent, rather than sequential, stimulus presentations (Fendrich and Corballis, 2001; Kitagawa and Ichihara, 2002; Repp and Penel, 2002; Bertelson and Aschersleben, 2003; Burr et al., 2009). Our results indicate that exposure to auditory stimuli can impact visual perception even when the different modalities are separated by up to several minutes. The persistence of the auditory influence across time suggests a mechanism that is internally mediated, rather than stimulus-driven. An interesting question that remains to be addressed is how much auditory exposure is needed to induce the visual beat perception. The current design always used two auditory modality sessions (5 min each) before testing the visual modality. However, as beat perception with auditory stimuli can occur very rapidly, it is possible that exposure to just a few auditory sequences would be enough to generate an internal representation that can subsequently induce visual beat perception.

The current behavioral results fit within the broader literature related to modality differences in timing. It is generally the case that the auditory modality has an advantage when it comes to processing temporal information. The temporal ventriloquism and auditory driving phenomena are familiar examples of the ability of auditory temporal information to alter perception in the visual modality (Shipley, 1964; Bertelson and Aschersleben, 2003; Wada et al., 2003). One explanation for the auditory advantage over visual timing is a cost associated with transforming visual temporal information into an auditory code (Collier and Logan, 2000). Guttman et al. (2005) tested this hypothesis using an interference paradigm in which participants made same-different judgments about pairs of rhythms that were presented alone or concurrently with a rhythm in the other modality. They found that same-different judgments for visual rhythm pairs were worse when presented concurrently with incongruent than congruent auditory rhythms (indicating that auditory information influences visual timing). Task-irrelevant visual information also reduced accuracy, but the decrement was larger for irrelevant auditory information (indicating that auditory information was harder to ignore). Finally, task-irrelevant incongruent auditory information was more disruptive when presented concurrent with the first visual rhythm in a pair than the second rhythm (indicating that the encoding stage was more vulnerable than the comparison stage). Guttman et al. (2005) concluded that temporal structure is automatically and obligatorily abstracted from visual rhythms and represented using an auditory code, such that even irrelevant auditory information is disruptive to visual temporal encoding. Contrary to the conclusion reached by Guttman et al., our findings suggest that auditory encoding of visual sequences is neither obligatory nor automatic, as visual test sequences showed very different patterns of tempo judgments from auditory sequences; see also McAuley and Henry (2010). However, the auditory recoding of visual sequences can be facilitated by exposure to auditory versions of the rhythms; tempo judgments for visual test sequences more closely resembled auditory judgments only when the auditory condition had been experienced first.

### fMRI results

#### Activation differences and similarities between modalities

We observed an expected modality difference in brain areas activated by the auditory and visual tasks. fMRI showed greater activity for auditory than visual sequences in bilateral superior temporal gyri, insula, basal ganglia, and cuneus. Activity in the superior temporal gyri is certainly expected in auditory tasks, and the insula is also routinely associated with auditory processing (Bamiou et al., 2003), particularly during discrimination of auditory temporal sequences and perception of rhythm (Griffiths et al., 1997; Platel et al., 1997; Thaut, 2003; Vuust et al., 2006). Especially relevant for the current study, Grahn and McAuley (2009) previously showed that the left insula was more active for individuals with strong beat perception (high *w*-values) than for individuals with weak beat perception (low *w*-values) in auditory stimuli identical to those used in the current study. As *w*-values were higher for the auditory than the visual modality, insula activation may reflect greater overall beat perception. Higher overall levels of beat perception would also be expected to activate the basal ganglia, as previous studies have shown greater basal ganglia activity during beat perception (Grahn and Brett, 2007; Grahn and Rowe, 2009), and that basal ganglia dysfunction also leads to poorer discrimination of rhythms with a regular beat (Grahn and Brett, 2009).

Cuneus activation for auditory rhythms is less expected; however, the cuneus is often less active during task performance than during rest (Gusnard and Raichle, 2001), and the activity for this region was baseline > auditory > visual, suggesting that the auditory condition was easier for participants, resulting in less task-induced deactivation. This interpretation is supported by the somewhat lower thresholds for the auditory than visual judgments.

Greater visual than auditory activity was found in bilateral occipital cortex, fusiform gyri, superior parietal cortex, and cerebellum. For the visual modality, the activation foci in occipital, parietal, and right inferior frontal areas closely match those observed by Jantzen et al. (2005) for tapping to a visual pacing signal compared to an auditory one. The authors suggested that these areas may be involved in visuomotor transformation processes, since a tapping response was required throughout the task. In the current study no production was required, suggesting a more general role in visual timing processes shared by perception and production. The cerebellum is implicated in timing and rhythm perception/production by both neuroimaging and neuropsychological work (Mangels et al., 1998; Penhune et al., 1998; Ivry et al., 2002; Grube et al., 2010), and the cerebellum has been argued to be a crucial component of timing in the milliseconds to seconds range (Lewis and Miall, 2003; Del Olmo et al., 2007), the same range that characterized our stimuli. However, counter to the view that the cerebellum comprises a supramodal timer (Ivry, 1997), we observed greater activation for the visual task than the auditory task in this area. Given higher thresholds in the visual task relative to the auditory task, this finding is consistent with an imaging study showing that cerebellum activity increases with increasing difficulty in timing tasks (Penhune et al., 1998).

We observed activations common to both modalities in bilateral inferior frontal cortex (BA 44), SMA, premotor cortex, posterior superior temporal gyri, supramarginal gyri (BA 40), putamen, and cerebellum. The overlap between modalities in these areas is in line with the previous research of Schubotz et al. (2000), which compared auditory and visual rhythm perception and found a similar network of brain areas activated across modalities. Our findings are also consistent with those of Karabanov et al. (2009), who examined influences of modality on rhythm production (rather than perception). In that study, auditory or visual rhythms were learned prior to scanning. During scanning, an auditory or a visual pacing signal cued participants to reproduce a learned rhythm. Reproduction of rhythms that were both trained and paced in the visual modality activated essentially the same areas as the reproduction of auditory rhythms (including SMA, premotor cortex, frontal operculum, posterior superior temporal gyri, cerebellum, and basal ganglia). One difference is that activations in our study and Schubotz et al. are generally more bilateral, perhaps because our tasks involved perception rather than reproduction (Hickok, 2001).

#### Auditory exposure influences on visual condition activity

A novel contribution of the current work concerns activation for visual sequences with and without prior auditory exposure (AV versus VA order). For the visual condition, there were robust increases for the AV order in bilateral putamen, as well as more circumscribed and less robust activations in right cerebellum, middle frontal gyrus, inferior temporal gyrus, and left inferior occipital gyrus. These activation changes accompanied a behavioral increase in visual beat sensitivity. In particular, as shown in Fig. 5, the activity in putamen and right cerebellum closely paralleled the order effect observed for the behavioral index of beat perception. That is, these areas showed significant activity during the auditory condition (compared to rest), regardless of order; similarly, behavioral data showed high beat sensitivity in both auditory conditions regardless of order. In the visual condition (compared to rest), only the AV order group showed activity in these regions; similarly, behavioral data revealed increased beat perception during the visual condition in the AV order only. To further explore the relationship between activation and behavior, a regression analysis predicting *w* values for visual test sequences using putamen and cerebellum activation was conducted. Putamen, but not cerebellar, activity predicted the beat sensitivity in visual test sequences. Thus, individual increases in beat sensitivity in the AV group are directly related to the activity levels in the putamen (but not in the cerebellum). Critically, we did not observe any areas of significantly increased activation during the visual condition for the VA order relative to the AV order.

One potential explanation for the observed modality by order interaction that needs to be considered is time in the scanner; the visual session was experienced later for the AV group than the VA group, meaning that changes in activation could be due to practice effects, learning, fatigue, etc., i.e., effects that are confounded with time. Although the AV group experienced the visual sessions later than the VA group, several analyses indicated that the areas described above did not correlate with time spent in the scanner. First, as shown in Supplementary Figure 1, activity in these areas tended to decrease over time, whereas *increases* were observed in the visual condition for the AV group compared to the VA group. Second, there were no differences in auditory condition activity between the VA and AV groups in these brain areas (shown in Fig. 5), even though the auditory condition in the VA group was experienced later than the AV group. Therefore, the interaction effects in the cerebellum and basal ganglia are due to the order in which the participants experienced the auditory and visual modalities, and do not show linear trends for increasing activity related to time in the scanner or doing the task. A related consideration is whether the observed order effects are specific to having performed that task in the auditory domain or would also have occurred if the participants performed the visual task for more time. The activation patterns for the two sessions of the visual task suggest that the order effects are specific to auditory influences, as activation decreased from the first to the second visual session. However, future research should investigate whether sensitivity to a beat in visual rhythms, and corresponding brain activation, can be increased by prolonged exposure to visual rhythms with an implied beat.

Activity in the putamen directly predicted increases in *w* values for the visual session after auditory exposure. The putamen is part of the striatum, which is known to play a crucial role in timing more generally. For example, impaired timing is found in disorders that affect the basal ganglia, such as Parkinson's disease (Artieda et al., 1992; Malapani et al., 1998, 2002) and Huntington's disease (Paulsen et al., 2004). Data from the neurophysiological domain also implicates the striatum, particularly oscillations in thalamo-cortico-striatal loops (Alexander et al., 1990; Matell et al., 2003). These findings have led to the formation of an influential model (the striatal beat-frequency model), which suggests that medium spiny neurons in the basal ganglia act as coincidence detectors for cortical neural oscillators (Matell and Meck, 2004; Buhusi and Meck, 2005). The coincidence detection of activity in different neural populations is suggested to underpin a variety of cognitive functions, including timing. Although the striatal beat frequency model has been proposed mainly for timing in the seconds to minutes range, oscillator approaches in the sub-second range have also proved to be a fruitful approach to modeling aspects of time and rhythm perception (Large and Kolen, 1994; McAuley and Kidd, 1998; Large, 2000; McAuley and Jones, 2003).

Empirically, a role for the basal ganglia in beat-based timing has already been shown in the auditory modality with fMRI and with Parkinson's disease patients (Grahn and Brett, 2007; Grahn and Brett, 2009). Here we show that the basal ganglia may even contribute to beat perception in the visual modality, particularly when beat perception is promoted by previous auditory exposure. Although a beat is not readily induced in the visual modality (Patel et al., 2005), we propose that when an internal representation of a beat is available because of prior auditory experience, this representation can be exploited to support beat perception in the visual modality. This view is consistent with the finding that greater basal ganglia activity is observed when internal generation of a beat is required (Grahn and Rowe, 2009). One way that increased visual beat perception could be accomplished is by using an internal beat representation to facilitate auditory imagery: after experiencing the auditory condition, participants can imagine the auditory tones occurring during the visual presentation of the sequence. We did not instruct participants to use auditory imagery, but some participants did report imagining sounds during the visual condition. This possibility is further supported by evidence that the basal ganglia are also involved in imagery (Dominey et al., 1995). Another potential mechanism is that an amodal beat representation, for example a motor representation, is created when listening to the auditory stimuli and can subsequently be accessed during either auditory or visual stimulus presentation. One way to evaluate these two possibilities would be to examine order effects for beat perception based on tactile exposure. Behaviorally, a beat is perceivable in the tactile modality (Brochard et al., 2008), and the basal ganglia have been shown to be active during temporal processing of somatosensory temporal stimuli (Pastor et al., 2004). This suggests that a tactile–visual exposure order should produce similar effects to the auditory–visual order. If the effects did occur for tactile–visual ordering, then an auditory imagery account of the current findings becomes less likely, and an explanation based on an amodal representation (or transformation into a motor representation) would be more fitting.

Our regression analysis did not find any significant predictive value of cerebellar activity for visual beat sensitivity. Cerebellum activation is often reported for timing tasks (Penhune et al., 1998; Rao et al., 2001; Pastor et al., 2004), but has not been shown to be specifically activated during beat perception in fMRI (Grahn and Brett, 2007). In addition, no evidence for a cerebellar role in beat perception has been found in neuropsychological patient work. Specifically, patients with cerebellar ataxia show no deficits in timing of rhythmic sequences based on a regular beat, but are significantly impaired only on timing tasks explicitly designed *not* to be based on a beat (e.g., comparing durations of two intervals) (Grube et al., 2010). Taken together, these findings suggest that the increased activation during visual sequences after auditory exposure seems unlikely to be related to beat perception. Several reports implicate the cerebellum in acquisition of sensory information and generation of motor output (Rao et al., 2001; Harrington et al., 2004), indicating a role for the cerebellum in sensorimotor transformation. It has been suggested that auditory rhythm information is automatically converted to a motor code (Halpern and Zatorre, 1999; Zatorre et al., 2007); increased cerebellum activation during visual sessions following auditory exposure may indicate that prior auditory exposure promotes visuo-motor transformation.

The only previous modality order effect of which we are aware was a deactivation of the left angular gyrus following auditorily–but not visually–paced rhythm reproduction (Karabanov et al., 2009). We found no modulation of angular gyrus activity, but our design blocked modality within sessions (as opposed to intermixing of auditory and visual stimuli, cf. Karabanov et al. (2009)), obscuring any transient effects occurring immediately after the modality switch. Though Schubotz et al. (2000) counterbalanced the order of presentation of auditory and visual sequences across participants, they did not report analyses of order-related differences.

## Conclusions

Prior auditory experience increases the likelihood of perceiving an implied beat in visual sequences. Moreover, an increase in visual beat perception is associated with an increase in basal ganglia activity. These results provide further evidence for a basal ganglia role in the internal generation of a beat and suggest that hearing an auditory rhythm creates an internal representation that encourages an auditory recoding of a subsequently presented visual rhythm.

## Figures and Tables

**Table 1:** Peak coordinates of the auditory–visual modality and visual–auditory modality.

Contrast	Brain region	Brodmann area	*t*	pFDR	*x*	*y*	*z*
Auditory–Visual							
	R inferior frontal gyrus p. triangularis	BA 45	3.75	0.002	54	36	0
	R anterior cingulate gyrus	BA 24	3.41	0.006	6	15	30
	R Rolandic operculum		14.89	<0.001	48	− 21	12
	L insula		3.79	0.002	− 39	9	9
			3.61	0.003	− 36	24	6
			3.40	0.007	− 39	15	− 9
			3.06	0.016	− 39	− 3	− 15
	L superior temporal gyrus	BA 22	13.00	<0.001	− 54	− 18	9
	BA 22	12.23	<0.001	− 57	− 33	12
		BA 22	7.78	<0.001	− 51	− 3	− 3
	R superior temporal gyrus	BA 22	14.98	<0.001	57	− 18	3
	BA 22	12.88	<0.001	51	− 9	0
	R superior temporal pole	BA 38	8.04	<0.001	54	3	− 6
	L cuneus	BA 17	6.15	<0.001	− 9	− 99	15
		BA 18	4.43	<0.001	− 9	− 93	30
	R cuneus	BA 18	5.94	<0.001	15	− 93	15
		BA 18	4.72	<0.001	9	− 78	30
	L calcarine sulcus	BA 19	4.37	<0.001	− 21	− 60	6
		BA 19	3.32	0.008	− 6	− 69	24
	R calcarine sulcus	BA 17	5.95	<0.001	6	− 72	6
		BA 17	5.89	<0.001	9	− 81	12
		BA 17	2.74	0.036	21	− 60	18
		BA 19	4.58	<0.001	27	− 57	6
	L superior occipital gyrus	BA 17	6.27	<0.001	− 6	− 75	3
	L lingual gyrus	BA 17	5.74	<0.001	− 12	− 87	9
		BA 18	5.16	<0.001	− 18	− 75	− 9
		BA 19	4.06	0.001	− 24	− 63	− 6
		BA 30	4.10	0.001	− 12	− 45	− 9
		BA 37	3.81	0.002	− 24	− 48	− 6
		BA 27	2.68	0.041	− 9	− 45	3
	R lingual gyrus	BA 17	5.67	<0.001	9	− 63	6
		BA 18	5.65	<0.001	12	− 60	3
		BA 18	5.38	<0.001	15	− 69	− 3
		BA 19	4.86	<0.001	21	− 60	− 6
	L fusiform gyrus	BA 37	3.71	0.003	− 30	− 54	− 3
		BA 30	2.94	0.022	− 21	− 42	− 18
	R fusiform gyrus	BA 37	5.19	<0.001	27	− 51	− 6
	L putamen		2.98	0.02	− 27	12	9
	R putamen		2.88	0.026	24	6	9
	L hippocampus		3.44	0.006	− 18	− 27	− 3
	R hippocampus		4.11	0.001	21	− 24	− 3
Visual–Auditory							
	R inferior frontal operculum	BA 44	5.86	<0.001	45	6	30
	L middle temporal gyrus	BA 37	11.17	<0.001	45	-66	6
	L superior occipital gyrus	BA 19	5.08	<0.001	− 24	− 78	27
	R middle occipital gyrus	BA 19	8.87	<0.001	30	− 72	27
	L middle occipital gyrus	BA 18	7.73	<0.001	− 30	− 87	0
	L inferior occipital gyrus	BA 19	10.53	<0.001	− 45	− 78	− 6
	BA 19	9.25	<0.001	− 39	− 84	− 6
	R inferior occipital gyrus	BA 19	9.59	<0.001	42	− 78	− 3
	BA 19	9.28	<0.001	33	− 84	0
	L fusiform gyrus	BA 19	7.36	<0.001	− 42	− 66	− 15
		BA 37	6.99	<0.001	− 42	− 57	− 15
	R fusiform gyrus	BA 37	7.05	<0.001	42	− 51	− 15
	L superior parietal lobule	BA 7	3.93	0.003	− 27	− 57	48
	R superior parietal lobule	BA 7	6.75	<0.001	30	− 51	48
	L cerebellum crus 1		3.61	0.007	− 45	− 63	− 36
	L cerebellum crus 2		4.75	<0.001	− 6	− 78	− 39
	R cerebellum crus 2		4.25	0.001	3	− 78	− 36

This table shows the brain region, Brodmann area, *t* values, pFDR values, and stereotaxic coordinates (in millimeters) of peak voxels. L: Left; R: right. Cluster size not given as clusters were large and contained many peaks from different anatomical areas.

**Table 2:** Peak coordinates of the conjunction between the visual and auditory modalities (pFDR < 0.05 whole-brain corrected).

Brain region	Brodmann area	*t*	pFDR	*x*	*y*	*z*
L inferior frontal gyrus p. triangularis	BA 45	4.51	<0.001	− 39	30	21
R inferior frontal gyrus p. opercularis	BA 46	5.62	<0.001	39	36	27
L inferior frontal operculum	BA 44	10.27	<0.001	− 45	6	24
	BA 44	6.34	<0.001	− 51	9	6
R inferior frontal operculum	BA 44	8.78	<0.001	51	9	21
L anterior cingulate gyrus	BA 32	3.17	0.011	− 15	27	24
	BA 32	4.22	<0.001	− 9	27	39
L supplementary motor area	BA 6	15.29	<0.001	− 3	3	60
	BA 6	16.27	<0.001	− 9	9	54
R supplementary motor area	BA 6	9.35	<0.001	6	15	48
L premotor cortex	BA 6	7.40	<0.001	− 27	− 6	48
	BA 6	11.33	<0.001	− 48	− 3	48
R premotor cortex	BA 6	7.57	<0.001	39	0	39
	BA 6	8.63	<0.001	54	0	45
	BA 6	7.17	<0.001	42	0	51
R precentral gyrus	BA 6	3.09	0.014	18	− 18	72
R paracentral lobule	BA 4	3.35	0.007	3	− 27	66
L postcentral gyrus	BA 4	8.19	<0.001	− 36	− 21	54
R Rolandic operculum		8.51	<0.001	54	12	15
L anterior insula	BA 47	8.49	<0.001	− 27	24	0
	BA 47	9.00	<0.001	30	24	0
L posterior superior temporal gyrus	BA 41	8.35	<0.001	− 48	− 42	21
R posterior superior temporal gyrus	BA 42	6.39	<0.001	57	− 39	15
	BA 22	6.82	<0.001	66	− 36	18
R middle temporal gyrus	BA 21	4.33	<0.001	42	− 42	9
R supramarginal gyrus	BA 40	6.97	<0.001	45	− 33	45
L inferior parietal lobule	BA 40	7.72	<0.001	− 36	− 45	39
	BA 40	5.79	<0.001	− 45	− 36	45
L middle occipital gyrus	BA 18	4.16	0.001	− 21	− 96	− 3
R middle occipital gyrus	BA 18	2.72	0.034	30	− 96	3
R inferior occipital gyrus	BA 18	3.34	0.007	21	− 90	− 12
	BA 19	3.66	0.003	33	− 84	− 9
L lingual gyrus	BA 19	4.00	0.001	− 33	− 84	− 12
R lingual gyrus	BA 18	3.43	0.005	24	− 90	− 6
L putamen		6.43	<0.001	− 18	3	3
R putamen		5.37	<0.001	18	6	− 3
R cerebellum lobule IV/V		3.05	0.015	12	− 48	− 21
L cerebellum lobule VI		7.67	<0.001	− 27	− 60	− 27
R cerebellum lobule VI		7.53	<0.001	30	− 60	− 27
R cerebellum lobule VIIb		3.85	0.002	18	− 75	− 45
L cerebellum lobule VIII		6.75	<0.001	− 24	− 69	− 51
R cerebellum lobule VIII		5.86	<0.001	33	− 63	− 51
R cerebellum crus 1		5.02	<0.001	42	− 66	− 27
L cerebellum crus 2		3.51	0.004	− 9	− 78	− 33
L thalamus		4.06	0.001	− 12	− 18	6
R thalamus		3.54	0.004	12	− 12	6
midbrain		4.83	<0.001	− 6	− 18	− 3
R midbrain		3.62	0.003	6	− 18	− 3

This table shows the brain region, Brodmann area, *t* values, pFDR values, and stereotaxic coordinates (in millimeters) of peak voxels. L: Left; R: right. Cluster size not given as clusters were large and contained several peaks from different anatomical areas.

**Table 3:** Areas showing a significant interaction between group and modality.

Brain region	Brodmann area	Cluster size (voxels)	*F*	pFDR	*x*	*y*	*z*
R putamen		14	22.3	0.022	18	9	− 6
L putamen		50	25.75	0.022	− 18	12	− 6
L caudate		10	22.53	0.022	− 18	12	15
R pallidum		2	19.61	0.026	18	6	9
R anterior insula/VLPFC	BA 47	10	20.73	0.022	33	30	21
L SMA	BA 6	2	19.51	0.026	− 9	9	57
R inferior temporal gyrus	BA 19	10	22.38	0.022	48	− 72	− 6
L inferior occipital gyrus	BA 19	10	25.28	0.022	− 48	− 78	− 3
R fusiform gyrus	BA 37	2	17.21	0.040	39	− 60	− 12

This table shows the brain region, Brodmann area, cluster size, *F* values, pFDR values, and stereotaxic coordinates (in millimeters) of peak voxels. L: Left; R: right.

**Table 4:** Areas with significantly greater visual condition activity in the auditory-first (AV) order compared to the visual-first (VA) order (pFDR < 0.05, whole-brain corrected, and masked by the F-contrast of the interaction between modality and order at *p* < 0.001 uncorrected to exclude non-specific order-related differences present in both modalities).

Brain Region	Brodmann Area	Cluster size (voxels)	*t*	pFDR	*x*	*y*	*z*
R putamen		37	5.13	0.007	18	9	− 6
L putamen		83	4.99	0.007	− 18	6	− 6
			4.42	0.012	− 27	18	0
R middle frontal gyrus	BA 46	2	3.89	0.03	33	30	21
R inferior temporal gyrus	BA 37	1	3.81	0.034	45	− 48	− 9
L inferior occipital gyrus	BA 19	6	4.52	0.01	− 48	− 78	− 3
R cerebellum, 8th lobule		4	3.95	0.026	36	− 57	− 45

This table shows the brain region, Brodmann area, cluster size, *t* values, pFDR values, and stereotaxic coordinates (in millimeters) of peak voxels. L, Left; R, right.

## References

[bb0005] Alexander G.E., Crutcher M.D., DeLong M.R. (1990). Basal ganglia-thalamocortical circuits: parallel substrates for motor, oculomotor, “prefrontal” and “limbic” functions. Prog. Brain Res..

[bb0010] Artieda J., Pastor M.A., Lacruz F., Obeso J.A. (1992). Temporal discrimination is abnormal in Parkinson's disease. Brain.

[bb0015] Bamiou D.-E., Musiek F.E., Luxon L.M. (2003). The insula (Island of Reil) and its role in auditory processing: literature review. Brain Res. Rev..

[bb0020] Bengtsson S.L., Ullén F., Henrik Ehrsson H., Hashimoto T., Kito T., Naito E., Forssberg H., Sadato N. (2009). Listening to rhythms activates motor and premotor cortices. Cortex.

[bb0025] Bertelson P., Aschersleben G. (2003). Temporal ventriloquism: crossmodal interaction on the time dimension. 1. Evidence from auditory–visual temporal order judgment. Int. J. Psychophysiol..

[bb0030] Brochard R., Touzalin P., Després O., Dufour A. (2008). Evidence of beat perception via purely tactile stimulation. Brain Res..

[bb0035] Buhusi C., Meck W.H. (2005). What makes us tick? Functional and neural mechanisms of interval timing. Nat. Rev. Neurosci..

[bb0040] Burr D., Banks M., Morrone M. (2009). Auditory dominance over vision in the perception of interval duration. Exp. Brain Res..

[bb0045] Chen J.L., Penhune V.B., Zatorre R.J. (2008). Listening to musical rhythms recruits motor regions of the brain. Cereb. Cortex.

[bb0050] Collier G.L., Logan G. (2000). Modality differences in short-term memory for rhythms. Mem. Cogn..

[bb0055] Coull J.T. (2004). fMRI studies of temporal attention: allocating attention within, or towards, time. Cogn. Brain Res..

[bb0060] Del Olmo M.F., Cheeran B., Koch G., Rothwell J.C. (2007). Role of the cerebellum in externally paced rhythmic finger movements. J. Neurophysiol..

[bb0065] Dominey P.F., Decety J., Broussolle E., Chazot G., Jeannerod M. (1995). Motor imagery of a lateralized sequential task is asymmetrically slowed in Parkinson's disease. Neuropsychologia.

[bb0070] Drake C., Jones M.R., Baruch C. (2000). The development of rhythmic attending in auditory sequences: attunement, referent period, focal attending. Cognition.

[bb0075] Fendrich R., Corballis P.M. (2001). The temporal cross-capture of audition and vision. Percept. Psychophys..

[bb0080] Ferrandez A.M., Hugueville L., Lehéricy S., Poline J.B., Marsault C., Pouthas V. (2003). Basal ganglia and supplementary motor area subtend duration perception: an fMRI study. Neuroimage.

[bb0390] Gault R.H., Goodfellow L.D. (1938). An empirical comparison of audition, vision, and touch in the discrimination of temporal patterns and ability to reproduce them. J. Gen. Psych..

[bb0085] Glenberg A.M., Jona M. (1991). Temporal coding in rhythm tasks revealed by modality effects. Mem. Cogn..

[bb0090] Glenberg A.M., Mann S., Altman L., Forman T. (1989). Modality effects in the coding and reproduction of rhythms. Mem. Cogn..

[bb0095] Goodfellow L.D. (1934). An empirical comparison of audition, vision, and touch in the discrimination of short intervals of time. Am. J. Psychol..

[bb0100] Grahn J.A., Brett M. (2007). Rhythm perception in motor areas of the brain. J. Cogn. Neurosci..

[bb0105] Grahn J.A., Brett M. (2009). Impairment of beat-based rhythm discrimination in Parkinson's disease. Cortex.

[bb0110] Grahn J.A., McAuley J.D. (2009). Neural bases of individual differences in beat perception. Neuroimage.

[bb0115] Grahn J.A., Rowe J.B. (2009). Feeling the beat: premotor and striatal interactions in musicians and non-musicians during beat processing. J. Neurosci..

[bb0120] Griffiths T.D., Jackson M.C., Spillane J.A., Friston K.J., Frackowiak R.S.J. (1997). A neural substrate for musical hallucinosis. Neurocase Case Stud. Neuropsychol. Neuropsychiatry Behav. Neurol..

[bb0125] Grondin S. (2001). Discriminating time intervals presented in sequences marked by visual signals. Percept. Psychophys..

[bb0130] Grondin S., McAuley D. (2009). Duration discrimination in crossmodal sequences. Perception.

[bb0135] Grondin S., Rousseau R. (1991). Judging the relative duration of multimodal short empty time intervals. Percept. Psychophys..

[bb0140] Grube M., Cooper F.E., Chinnery P.F., Griffiths T.D. (2010). Dissociation of duration-based and beat-based auditory timing in cerebellar degeneration. Proc. Natl Acad. Sci. USA.

[bb0145] Gusnard D.A., Raichle M.E. (2001). Searching for a baseline: functional imaging and the resting human brain. Nat. Rev. Neurosci..

[bb0150] Guttman S.E., Gilroy L.A., Blake R. (2005). Hearing what the eyes see: auditory encoding of visual temporal sequences. Psychol. Sci..

[bb0155] Halpern A.R., Zatorre R.J. (1999). When that tune runs through your head: a PET investigation of auditory imagery for familiar melodies. Cereb. Cortex.

[bb0160] Harrington D.L., Haaland K.Y. (1999). Neural underpinnings of temporal processing: a review of focal lesion, pharmacological, and functional imaging research. Rev. Neurosci..

[bb0165] Harrington D.L., Haaland K.Y., Hermanowitz N. (1998). Temporal processing in the basal ganglia. Neuropsychology.

[bb0170] Harrington D.L., Lee R.R., Boyd L.A., Rapcsak S.Z., Knight R.T. (2004). Does the representation of time depend on the cerebellum. Brain.

[bb0175] Hickok G. (2001). Functional anatomy of speech perception and speech production: psycholinguistic implications. J. Psycholinguist. Res..

[bb0180] Ivry R. (1997). Cerebellar timing systems. Int. Rev. Neurobiol..

[bb0185] Ivry R., Keele S.W. (1989). Timing functions of the cerebellum. J. Cogn. Neurosci..

[bb0190] Ivry R.B., Spencer R.M., Zelaznik H.N., Diedrichsen J., Highstein S.M., Thach W.T. (2002). The cerebellum and event timing. New directions in cerebellar research.

[bb0195] Jantzen K.J., Steinberg F.L., Kelso J.A.S. (2005). Functional MRI reveals the existence of modality and coordination-dependent timing networks. Neuroimage.

[bb0200] Jongsma M.L.A., Quiroga R.Q., van Rijn C.M. (2004). Rhythmic training decreases latency-jitter of omission evoked potentials (OEPs) in humans. Neurosci. Lett..

[bb0205] Karabanov A., Blom Ö., Forsman L., Ullén F. (2009). The dorsal auditory pathway is involved in performance of both visual and auditory rhythms. Neuroimage.

[bb0210] Kitagawa N., Ichihara S. (2002). Hearing visual motion in depth. Nature.

[bb0215] Kolers P.A., Brewster J.M. (1985). Rhythm and responses. J. Exp. Psychol. Hum. Percept. Perform..

[bb0220] Large E.W. (2000). On synchronizing movements to music. Hum. Mov. Sci..

[bb0225] Large E.W., Kolen J.F. (1994). Accent structures in music performance. Connect. Sci..

[bb0230] Lewis P.A., Miall R.C. (2003). Distinct systems for automatic and cognitively controlled time measurement: evidence from neuroimaging. Curr. Opin. Neurobiol..

[bb0235] Llamon W.T., Goldstone S. (1974). Studies of auditory–visual differences in human time judgment. 2. More transmitted information with sounds than lights. Percept. Mot. Skills.

[bb0240] Macar F., Lejeune H., Bonnet M., Ferrara A., Pouthas V., Vidal F., Maquet P. (2002). Activation of the supplementary motor area and of attentional networks during temporal processing. Exp. Brain Res..

[bb0245] Malapani C., Rakitin B., Levy R., Meck W.H., Deweer B., Dubois B., Gibbon J. (1998). Coupled temporal memories in Parkinson's disease: a dopamine-related dysfunction. J. Cogn. Neurosci..

[bb0250] Malapani C., Deweer B., Gibbon J. (2002). Separating storage from retrieval dysfunction of temporal memory in Parkinson's disease. J. Cogn. Neurosci..

[bb0255] Mangels J.A., Ivry R.B., Shimizu N. (1998). Dissociable contributions of the prefrontal and neocerebellar cortex to time perception. Cogn. Brain Res..

[bb0260] Matell M.S., Meck W.H. (2004). Cortico-striatal circuits and interval timing: coincidence detection of oscillatory processes. Cogn. Brain Res..

[bb0265] Matell M.S., Meck W.H., Nicolelis M.A. (2003). Interval timing and the encoding of signal duration by ensembles of cortical and striatal neurons. Behav. Neurosci..

[bb0270] McAuley J.D., Henry M.J. (2010). Modality effects in rhythm processing: auditory encoding of visual rhythms is neither obligatory nor automatic. Atten. Percept. Psychophys..

[bb0275] McAuley J.D., Jones M.R. (2003). Modeling effects of rhythmic context on perceived duration: a comparison of interval and entrainment approaches to short-interval timing. J. Exp. Psychol. Hum. Percept. Perform..

[bb0280] McAuley J.D., Kidd G.R. (1998). Effect of deviations from temporal expectations on tempo discrimination of isochronous tone sequences. J. Exp. Psychol. Hum. Percept. Perform..

[bb0285] McAuley J.D., Frater D., Janke K., Miller N.S. (2006). Detecting changes in timing: evidences for two modes of listening. The Proceedings of the 9th International Conference on Music Perception and Cognition.

[bb0290] McAuley J.D., Jones M.R., Holub S., Johnston H.M., Miller N.S. (2006). The time of our lives: life span development of timing and event tracking. J. Exp. Psychol. Gen..

[bb0295] Nenadic I., Gaser C., Volz H.-P., Rammsayer T., Hager F., Sauer H. (2003). Processing of temporal information and the basal ganglia: new evidence from fMRI. Exp. Brain Res..

[bb0300] Parncutt R. (1994). A perceptual model of pulse salience and metrical accent in musical rhythms. Music Percept..

[bb0305] Pastor M.A., Day B.L., Macaluso E., Friston K.J., Frackowiak R.S.J. (2004). The functional neuroanatomy of temporal discrimination. J. Neurosci..

[bb0310] Patel A.D., Iversen J.R., Chen Y., Repp B.H. (2005). The influence of metricality and modality on synchronization with a beat. Exp. Brain Res..

[bb0315] Paulsen J.S., Zimbelman J.L., Hinton S.C., Langbehn D.R., Leveroni C.L., Benjamin M.L., Reynolds N.C., Rao S.M. (2004). fMRI biomarker of early neuronal dysfunction in presymptomatic Huntington's disease. AJNR Am. J. Neuroradiol..

[bb0320] Penhune V.B., Zatorre R.J., Evans A.C. (1998). Cerebellar contributions to motor timing: a PET study of auditory and visual rhythm reproduction. J. Cogn. Neurosci..

[bb0325] Penny W., Holmes A.P., Frackowiack R.S.J., Friston K.J., Frith C.D., Dolan R., Price C.J., Ashburner J., Penny W., Zeki S. (2003). Random Effects Analysis. Human Brain Function II.

[bb0330] Platel H., Price C., Baron J.-C., Wise R., Lambert J., Frackowiak R.S.J., Lechevalier B., Eustache F. (1997). The structural components of music perception: a functional anatomical study. Brain.

[bb0395] Povel D.J., Essens P.J. (1985). Perception of temporal patterns. Music Perception.

[bb0335] Rao S.M., Mayer A.R., Harrington D.L. (2001). The evolution of brain activation during temporal processing. Nat. Neurosci..

[bb0340] Rencanzone G.H. (2002). Auditory influences on visual temporal rate perception. J. Neurophysiol..

[bb0345] Repp B.H., Penel A. (2002). Auditory dominance in temporal processing: new evidence from synchronization with simultaneous visual and auditory sequences. J. Exp. Psychol. Hum. Percept. Perform..

[bb0350] Schubotz R.I., von Cramon D.Y. (2001). Interval and ordinal properties of sequences are associated with distinct premotor areas. Cereb. Cortex.

[bb0355] Schubotz R.I., Friederici A.D., von Cramon D.Y. (2000). Time perception and motor timing: a common cortical and subcortical basis revealed by fMRI. Neuroimage.

[bb0360] Shih, L.Y.L., Kuo, W.-J., Yeh, T.-C., Tzeng, O.J.L., Hsieh, J.-C., 2009. Common neural mechanisms for explicit timing in the sub-second range. NeuroReport 20:897-901 810.1097/WNR.1090b1013e3283270b3283276e. 10.1097/WNR.0b013e3283270b6e 19451837

[bb0365] Shipley T. (1964). Auditory flutter-driving of visual flicker. Science.

[bb0400] Snyder, J.S., Pasinski, A., McAuley, J.D., in press. Listening strategy modulates cognitive processing of auditory rhythms. Psychophysiology. 10.1111/j.1469-8986.2010.01053.x 20557484

[bb0370] Thaut M.H. (2003). Neural basis of rhythmic timing networks in the human brain. Ann. NY Acad. Sci..

[bb0375] Vuust P., Roepstorff A., Wallentin M., Mouridsen K., Ostergaard L. (2006). It don't mean a thing... Keeping the rhythm during polyrhythmic tension, activates language areas (BA47). Neuroimage.

[bb0380] Wada Y., Kitagawa N., Noguchi K. (2003). Audio-visual integration in temporal perception. Int. J. Psychophysiol..

[bb0385] Zatorre R.J., Chen J.L., Penhune V.B. (2007). When the brain plays music: auditory-motor interactions in music perception and production. Nat. Rev. Neurosci..

