# Supplementary material for: FMRI investigation of cross-modal interactions in beat perception: Audition primes vision, but not vice versa
Source: Neuroimage. 2011 Jan 15;54(2-6):1231–43. doi: 10.1016/j.neuroimage.2010.09.033 (PMC3002396; doi:10.1016/j.neuroimage.2010.09.033)

Supplementary Methods: Model

To quantify individual differences in responses to the test sequences, we applied a signal detection model to the behavioral judgment response proportions. Binary (‘speeding up’ / ‘slowing down’) judgments were based on two temporal referents: P = 300 ms corresponding to the explicit time interval marked by the first three tones of the sequences and P = 600 ms corresponding to the implied beat. We then calculated, for each final interval of the sequence, *T_i_*, a temporal contrast metric, *C_i_*, which measured the normalized difference between the final interval and each referent, *P*:

$$C_{i}=\frac{(T_{i}-P)}{P}$$

In previous work, we’ve shown that the temporal contrast metric is a good index of the information that participants use to make time judgments decisions (McAuley and Jones, 2003). Because there were two possible temporal referents, each final interval, T_i_, resulted in two values of C_i_, labeled here as C_i300_ for the P = 300 ms referent and C_i600_ for the P = 600 ms referent. In line with standard signal detection assumptions (Macmillan and Creelman, 1991), values of temporal contrast for each referent were assumed to be normally distributed with standard deviation, σ; the values of C_i300_ and C_i600_ were then z-transformed and combined using a simple weighted average:

$$z=(1-w)z_{i_{300}}+wz_{i_{600}}$$

Predicted proportions of ‘speeding up’ responses, P(‘Speeding Up’), for each final interval, T_i_, were then generated using cumulative normal distribution function:

P(‘Speeding Up’) = 1 − Φ(z)

To fit the model to data, we allowed both *w* ∈ [0, 1] and *σ* to vary and minimized the root-mean-square error (RMSE) between the observed and predicted response proportions. Estimates of *w* were used as an index of beat-perception strength, while estimates of *σ* provided a separate index of temporal sensitivity. Based on the formulation of the model, values of *w* closer to 1 indicated greater sensitivity to the implied 600-ms beat.

Supplementary Figure 1. Mean signal intensity extracted from the brain regions showing significant differences in visual condition activity between groups (AV order and VA order), separated by session. The graphs indicate that the differences in visual condition activity between the AV and VA orders are not simply due to differences in the time spent in the scanner, as the activations are generally highest for the first visual session in the AV order, rather than the second.


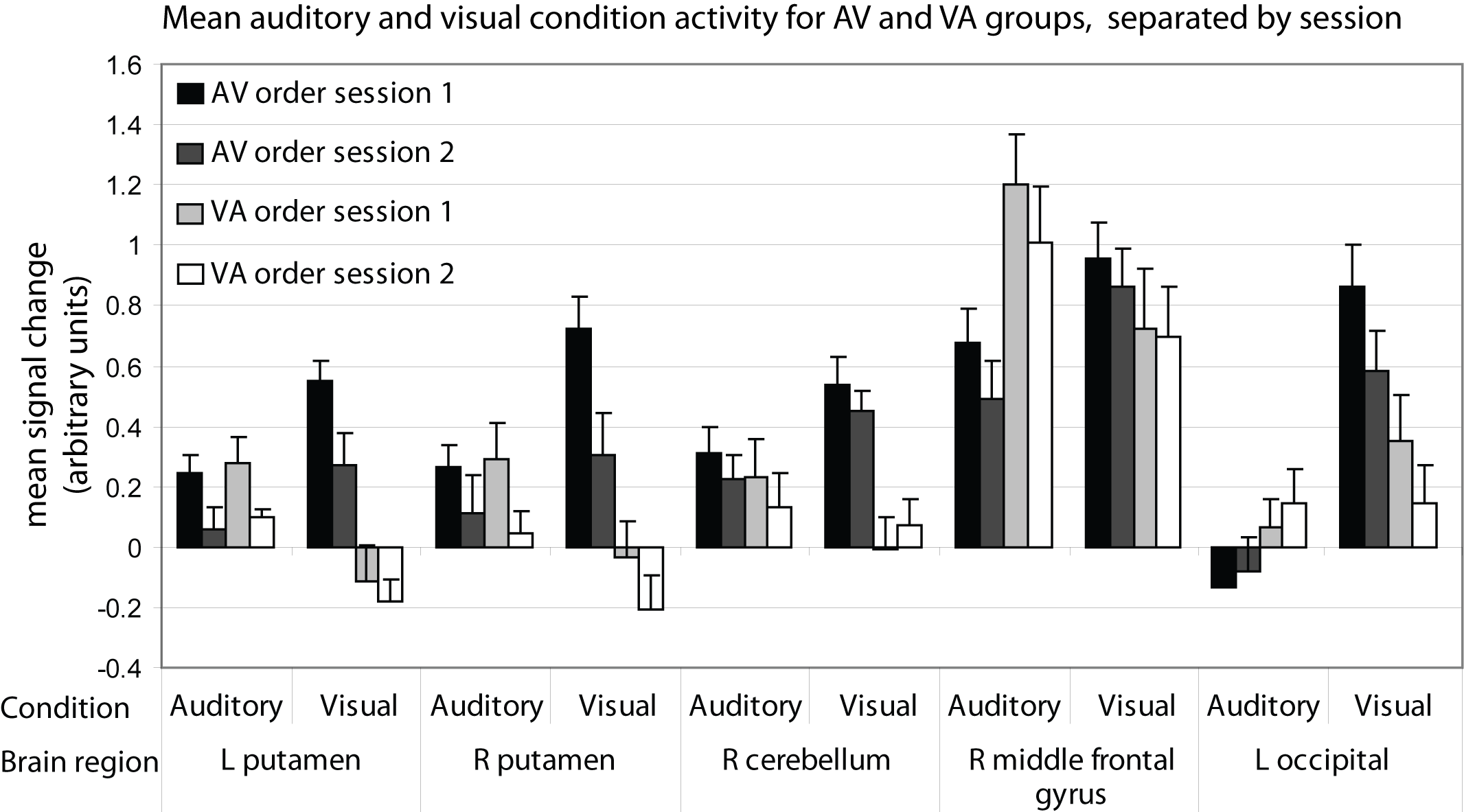

Supplement: Supplementary file 1 — Supplementary materials [file mmc1.doc]
